# Effect of ZnCl_2_ Treatment Parameters on the Thermo-Hydrolysis of Recycled MDF for Epoxy Composites

**DOI:** 10.3390/polym17182493

**Published:** 2025-09-15

**Authors:** Çağrı Olgun, Koray Çufa

**Affiliations:** 1 Department of Forest Industry Engineering, Faculty of Forestry, Kastamonu University, Orgeneral Atilla Ateş Paşa Cd., Emirler, Kastamonu Merkez, Kastamonu 37210, Türkiye; 2 Institute of Science, Department of Forest Industrial Engineering Kastamonu, Kastamonu University, Kastamonu 37150, Türkiye; koraycufa41@gmail.com; 3 Department of Impregnation Paperline, Kastamonu OSB. Plant, Kastamonu Entegre A.Ş., Kastamonu 37210, Türkiye

**Keywords:** MDF recycling, hydrothermal hydrolysis, zinc chloride, fiber characteristics epoxy composites

## Abstract

The aim of this study is to determine the hydrothermal recycling of medium-density fiberboard (MDF) wastes using zinc chloride (ZnCl_2_) as an acidic catalyst to obtain reinforcing fibers for epoxy-based composites. For this purpose, during the hydrothermal recycling process (110 °C, 0.4 bar), zinc chloride solutions with different concentrations (0% to 30%) were applied at different time intervals (20 to 60 min). The recycled fibers were characterized by Fourier transform infrared spectroscopy (FT-IR), scanning electron microscope-energy dispersive spectrometry (SEM-EDS), carbon (C) (%), hydrogen (H) (%), and nitrogen (N) (%) contents, and fiber classification. The fibers were added as a filler (1% *w*/*w*) to epoxy composites. The compression strength and of the epoxy composites as assessed and differential scanning calorimetry (DSC) characterization was performed. According to the results, nitrogen content decreased with increasing ZnCl_2_ concentration. Furthermore, the fine fibers ratios increased with increasing treatment time. The results suggest that recycled fibers can be used as a filler in epoxy composites; however, a long treatment time adversely affects the compression strength of epoxy composites.

## 1. Introduction

Considering the limited existing resources worldwide, an increasing population and rising consumption rates have increased the need for renewable raw materials today. Wood-based composites (such as particleboard, oriented strand board (OSB), and medium-density fiberboard (MDF)) play a critical role in meeting this demand [1]. In particular, MDF is the one of the most widely used raw materials in the global furniture industry and is also utilized in the construction sector due to its superior mechanical properties, including bending strength, internal bond strength, and modulus of elasticity, compared to particleboard [2,3].

MDF boards are generally produced with urea formaldehyde (UF), a synthetic thermosetting adhesive. After curing is complete, urea formaldehyde forms a cross-linked network structure that is insoluble, non-melted, and cannot be transformed [4]. This situation constitutes the most important issue in the disposal, storage in landfill areas and recycling of MDF’s at the end of their service life [5,6].

Various methods have been explored for recycling and recovery of MDF waste, and thermal hydrolysis is one of the most widely researched [7,8,9]. UF is susceptible to hydrolysis; therefore, hydrolysis conditions need to be optimized [10]. The degradation of UF by hydrolysis is influenced by its chemical structure and the degree of cross-linking; furthermore, this degradation can be accelerated by high temperatures and acidic conditions [11].

In hydrothermal hydrolysis recycling, it is essential to sever the binding between the urea-formaldehyde matrix and wooden fibers [7,12,13]. Acidic conditions are more effective than neutral and basic conditions in removing UF from fibers according to the literature [10,14]. However, during the process, deterioration in the fibers usually occurs due to the effects of temperature, pressure, and chemical agents [1,9,13,15,16,17]. Consequently, the length of recycled fibers generally decreases [1,5,13,18,19]. This situation is negatively impacts the mechanical performance of reconstituted boards and composites [15,20,21].

In addition, zinc chloride, ZnCl_2_, is a chemical known for its ability to induce cellulose swelling and is frequently used in the derivatization and hydrolysis of cellulose. Zn^2+^ ions, in particular, are known to penetrate fibers and cause them to swell [22]. Therefore, zinc chloride used in the hydrothermal hydrolysis process has potential for application in degrading UF and recovering MDF fibers. Although zinc chloride has been applied to biomass fractions for various purposes [22], its recycling application has been insufficiently researched.

Epoxy composites have established a significant presence in various application areas due to their advantages compared to other polymers. In addition, lignocellulosic fibers in particular are the most interesting ways to improve the properties of epoxy composites [23]. However, factors such as the addition rate, volume, and length of lignocellulosic fibers have significant effects on the reinforcement of the physical and mechanical properties of the composite to which they are added [24,25]. It is known that even adding 2–3% of lignocellulosic raw materials to epoxy composites results in a significant increase in their mechanical properties, especially in tensile strength, as is the focus in all epoxy studies [26,27,28,29,30]. Optimum values can be achieved at lower ratios, where wood fibers provide better reinforcement in plastic matrices than wood dust [31]. Fayzulla (2025) determined that a 5% addition of MDF fiber was optimal and used a minimum of 2% [28]. In another study, Das et al. (2024) incorporated kapok fiber into epoxy composites at loadings ranging from 1% to 7% and stated that mechanical values improved progressively from 1% to 5%, with the 5% value providing the optimum performance, while a further increase to 7% led to a decline [32]. Therefore, it is understood that this ratio can be further reduced, and recycled MDF fibers have great potential for use in this field.

The aim of this study is to investigate the effect of zinc chloride on the fibers when used at different concentrations and application times in MDF recycling by hydrothermal hydrolysis and to investigate the changes in some properties of epoxy composites (compression resistance, which is especially important in many areas of use, excluding many epoxy works, and heat resistance with DSC analysis) obtained from these fibers for sustainable and responsible production.

## 2. Materials and Methods

### 2.1. Material

Medium-density fiberboard (MDF) samples known to be produced using urea formaldehyde glue were provided as waste pieces. MDF samples were cut into strips of 1 cm width and 2–4 cm length to prepare them for recycling [9,20]. Analytical-grade zinc chloride was supplied from Balmumcu Kimya (Istanbul/Turkey) and used for the recycling procedures used in the study. The two-component ultra transparency epoxy resin (Stonemix) was supplied from Stone Kimya (Istanbul/Turkey).

### 2.2. Methods

#### 2.2.1. Recycling

The experimental design of the recycling processes is given in Table 1. Hydrothermal hydrolysis was applied for recycling of MDF samples. The samples were added to zinc chloride aqueous solutions with concentrations of 0%, 10%, 20%, and 30% (*w*/*w*%), using a solution/MDF waste ratio of 10:1. The mixtures were treated in an autoclave at 110 °C and 0.4 bar pressure for 20, 40, and 60 min. After the waste MDF samples had swollen and lost their integrity, the obtained fibers were washed with distilled water and mechanically dispersed. The fibers were then dried under room conditions (20 ± 2 °C and 65 ± 5% relative humidity) and occasionally mechanically stirred to prevent aggregation; some of the samples were oven dried at 103 ± 2 °C for the fiber analyses (except particle size distribution) to be carried out.

#### 2.2.2. Recycling Fiber Qualities

The recycled MDF fibers particle size distributions were determined with a sieve shaker (Retsch AS 200, Retsch, Haan, Germany). Approximately 5 g of oven-dried recycled fibers from each group was placed in the shaker, equipped with 10, 20, 30, 40, 60, 80, and 120 mesh screens (2000, 850, 600, 425, 250, 180, and 125 µm). The samples were shaken at 60 amplitude for 10 min. After classified samples were weighed, the percentage of each class was calculated according to the first sample weight ratio.

The obtained fiber samples were analyzed with Fourier transform infrared spectroscopy (FT-IR) to determine the differences in their functional groups. FT-IR experiments were carried out from 400 to 4000 cm^−1^ wavelengths with the help of the FT-IR ATR spectrophotometer (Bruker Alpha) located in the Central Research Laboratory of Kastamonu University.

Elemental analysis of carbon (C), hydrogen (H), and nitrogen (N) contents in the recycled fibers was carried out using a Eurovector CHN Elemental Analyzer at the Central Research Laboratory of Kastamonu University.

A Quanta FEG 250 (FEI, Hillsboro, OR, USA) scanning electron microscope (SEM) located in the Central Research Laboratory of Kastamonu University was used to determine the elemental composition. Scanning of the fiber surfaces was carried out with the help of energy dispersive spectrometry (SEM-EDS), focused on only C (%), O (%), and N (%).

#### 2.2.3. Epoxy Composites and Their Properties

Composites were created by adding 1% (*w*/*w*) recycled fiber dried at room conditions to two-component casting epoxy resin (component a:b 2:1 *w*/*w*). The mixture was put in a ultrasonic water bath (KUDOS, Shanghai, China) at 35 kHz for 10 min to provide a homogeneous composite. Then, the mixture was divided into molds with dimensions of 1.27 × 1.27 × 2.54 cm. The composites were allowed to harden completely (curing time: 48 h according to user guide) at room conditions

The density of epoxy composites was determined using the gravimetric method. Measurements were conducted on six samples for each group. The measurements (thickness, width, and length) were carefully taken using a digital caliper to determine the volume, and the mass was determined. The density (ρ) was determined using the subsequent Equation (1):

(1) $$\rho \;=\;\frac{m}{V}$$

where *m* is the sample mass and *V* is the calculated volume.

After determining the densities of the composite materials, their compressive strengths were determined according to ASTM D 695-15 [33] using a Shimadzu AG-IC 20/50 KN STD Universal Testing Machine (Kyoto, Japan) located in the Forestry Industry Laboratory of Kastamonu University, Faculty of Forestry.

Differential scanning calorimetry (DSC) analyses were performed according to EN ISO 11357-1 (2023) [34] at the Central Research Laboratory of Kastamonu University using a HITACHI DSC7020. Approximately 6 to 10 mg of epoxy composite samples for DSC were weighed into standard aluminum crucibles. Heating was performed from 0 to 250 °C for DSC at a rate of 5 °C min^−1^ under a nitrogen atmosphere.

## 3. Results and Discussion

### 3.1. Recycled Fiber Characterization

#### 3.1.1. Particle Size Distributions

**Figure 1:**
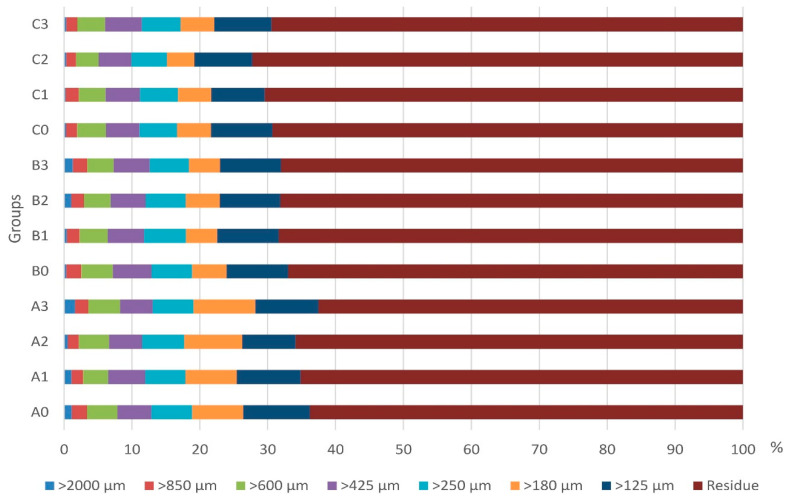
The particle size distributions of recycled fibers (treatment times: A = 20 min, B = 40 min, C = 60 min; ZnCl_2_ concentrations: 0 = 0%, 1 = 10%, 2 = 20%, 3 = 30%).

The particle size distributions of recycled fibers obtained from hydrothermal hydrolyzed MDF samples are shown in Figure 1. The fibers represented in Figure 1 can be roughly classified as fine (<250 µm), normal (250 µm ≤ x < 850 µm), and coarse (≤850 µm), similar to the classification used by Troilo et al. (2023) [17]. In general, after hydrothermal treatment, a significant increase in the amount of fine fibers was observed. In addition, it is understood from Figure 1 that the amount of fine fibers generally increased with the increase in chemical concentration and processing time. The main reason for the higher ratio fine fibers is that MDF fibers are first produced by thermo-mechanical processes including high temperature and pressure, then compressed under high temperature and pressure in the hot pressing during board formation, and lastly treated at high temperature and pressure again during recycling by hydrothermal hydrolysis [2]. It was reported in another study that the average fiber length of hydrothermal hydrolysis recycled fibers was 12.44% shorter than that of virgin fibers [1]. Similarly, it was reported that the amount of fine fibers in MDF fibers recycled by steam explosion is higher than in normal fibers [35]. Moezzipor et al. (2018) stated that the ratios of fine fibers recycled by electrical heating were higher than those of normal fibers [13]. This situation is negative for MDF remanufacturing, although it gives a considerable advantage for epoxy composites. Potadar and Kadam (2018) indicated that as the particle size of the biomass fibers increases from 1 mm to 2 mm, the tensile strength of epoxy composites decreases [36]. In addition, Chauhan et al. (2021) indicated in their study that tensile strength decreases dramatically when particle size reduces from 600 microns to 300 microns [37].

#### 3.1.2. FT-IR Analysis of Recycled Fibers

**Figure 2:**
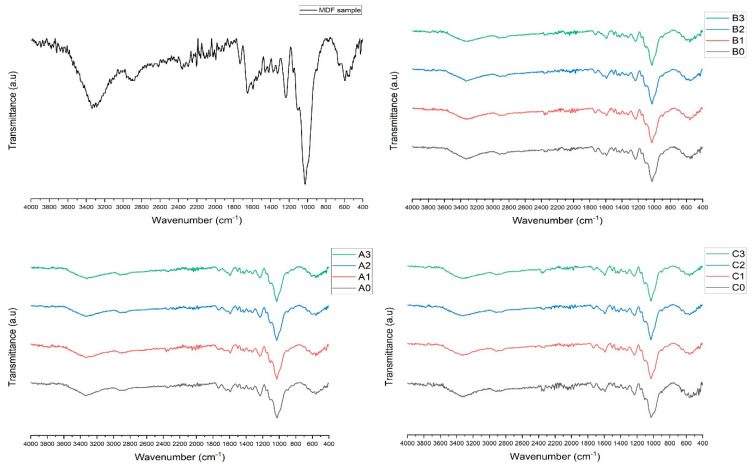
FT-IR spectrums of analyzed samples (treatment times: A = 20 min, B = 40 min, C = 60 min; ZnCl_2_ concentrations: 0 = 0%, 1 = 10%, 2 = 20%, 3 = 30%).

In Figure 2, FT-IR analysis graphs of the waste MDF sample and the samples obtained by the hydrothermal hydrolysis method are shown as transmittance ratio (T%). In the FT-IR analysis of the recycled fibers compared to the MDF sample in Figure 2, it was observed that there were significant differences in the wavelengths representing the C=O aliphatic aldehyde (formaldehyde)-induced stretching (1740–1720 cm^−1^), the C=O stretching band (Amide I) (1680–1630 cm^−1^), and finally the mixed C-N stretching and N-H bending (Amide II) (1600–1550 cm^−1^), especially in the region originating from urea formaldehyde [38]. Particularly, the most significant differences were clearly observed at Amide I (1648 cm^−1^ based on MDF sample FT-IR spectrum) and Amide II (1591 cm^−1^ based on MDF sample FT-IR spectrum). Similar to our study, recycling studies in the literature also reported that the greatest differences in FT-IR analyses were observed at these two wavelengths [2,9,14,39]. Therefore, the decrease in these two bonds can be considered as an indication that hydrothermal hydrolysis was successful in degrading the matrix structure of UF.

#### 3.1.3. Elemental Analysis of Recycled Fibers

Table 2 illustrates the results of the elemental analysis of recycled fibers. The amount of decrease in nitrogen is proportionally directly related to the amount of urea formaldehyde removed from the board [10,14,17,39]. In general, it was observed that the hydrothermal treatment duration did not affect the change in the total nitrogen amount from Table 2. While the highest percentage nitrogen amount for all treatment times was obtained from 0% concentration, the best values were obtained from the groups treated with ZnCl_2_ at 30% concentration. In addition, it was determined that the nitrogen content of the groups treated with ZnCl_2_ at 20% concentration were close to and effective to those treated with 30% concentration. According to the literature, the main reason for the changes in Table 2 is that the methylene and methylene ether bridges found in urea formaldehyde are hydrolyzed by water during thermo-hydrolysis [12,40]. It is known that urea formaldehyde removal can be accelerated by thermo-hydrolysis with the aid of acid [10,14]. It is likely that ZnCl_2_ dissolves in water and, in the acidic environment it creates, Zn^+2^ ions target the urea formaldehyde structure, accelerating its decomposition by water. Additionally, ZnCl_2_ is used in the literature for various purposes to swell cellulose [22]. Therefore, the swelling of cellulose during thermo-hydrolysis may facilitate the separation of fiber and adhesive bonds.

Lubis et al. (2018) reported that the nitrogen content in hot water recycling is 1.04%, and this value is decreased to 0.26% with the addition of sulfuric acid, but this percentage increased when base was added [14]. It has been reported that the nitrogen content in chips obtained by hydrothermal recycling of particleboard with only water decreases by 1.3% after 1 h of waiting at 100 °C [41]. It was stated by Buschalsky and Mai (2021) that the nitrogen content of the fibers obtained as a result of the recycling process applied to fiberboards of different thicknesses at different solution/board ratios and times at 95 °C varied between 1.0% and 2.5% [15]. In another study, it was stated that the nitrogen percentage of MDF waste decreased from 4.15% to 0.40% when the fibers were recycled with hot pressurized water (at 180 °C for 15 min) [17]. Similarly, Olgun (2025) determined the nitrogen content of hydrothermal recycled fiber as 0.924% after 30 min treatment with pure water [9]. In another study, it was found that the nitrogen ratio could be reduced to values close to those of crude fiber by using oxalic acid during hydrothermal hydrolysis [42]. Therefore, it can be easily said that the most likely reason for the effectiveness at 20% and 30% concentrations is that zinc chloride solutions provide an acidic pH.

#### 3.1.4. Morphological and Surface Analysis

**Figure 3:**
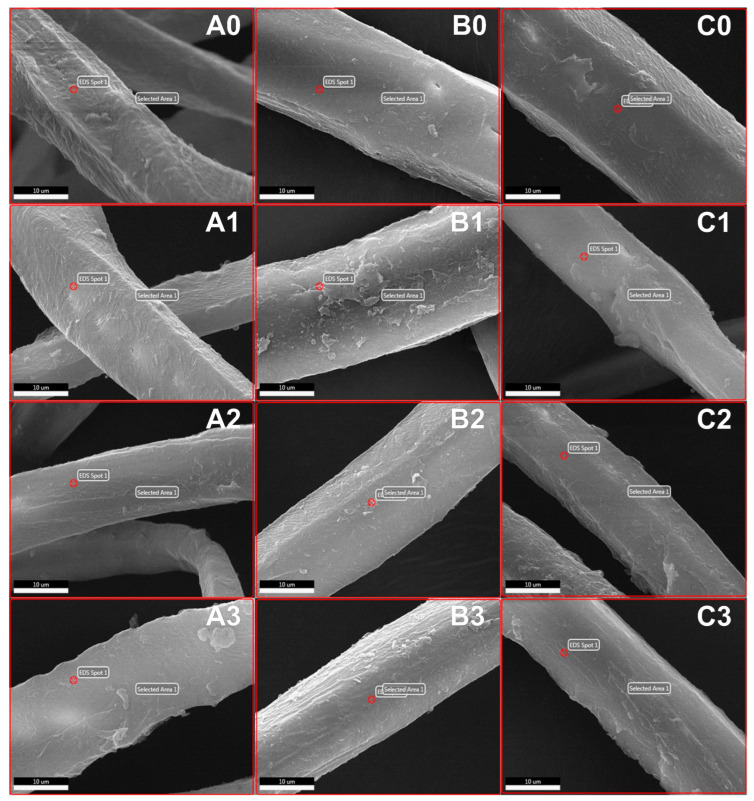
SEM photographs of recycled MDF fibers (treatment times: A = 20 min, B = 40 min, C = 60 min; ZnCl_2_ concentrations: 0 = 0%, 1 = 10%, 2 = 20%, 3 = 30%; cross in red circle: shows selected point or surface).

In the SEM photographs in Figure 3, adhesive residue is observed on the surfaces of all recycled fibers. The extent of morphological deformations, such as narrowing and a rough surface, in the fibers increases with higher concentration and longer processing time. In particular, narrowing of the fiber cross-sections can be observed in fibers recycled with ZnCl_2_ solution, such as samples A3 and C1. The main reason for this is the deformations caused by acid treatment in lignocellulosic fibers [43]. The increase in the amount of fine fibers in Figure 1 is one of the most basic indicators of this situation. Additionally, in Figure 3, the cell walls lose their surface smoothness, particularly as the concentration increases and the adhesive deposits on the fiber surfaces decrease. This also indicates that the acidic impact of zinc chloride strengthens with increasing concentration.

The adhesive residues remaining on the surface of the fibers after the recycling process hinder the reuse of the recovered fibers in the production of fiberboards. Even in very small amounts, adhesive residue on the wood surface can significantly reduce bonding strength [12,17]. Therefore, elemental analysis alone is often insufficient, as it also includes the nitrogen content inherently present in the recovered fibers. Residual adhesive on the fiber surface can be more precisely identified using Energy Dispersive Spectroscopy (EDS), which allows for the detection of elements in relation to the fibers’ surface area [9,17,44].

The elemental composition of the surface areas of the recycled fibers obtained in the study, along with the weight and atomic percentages of the elements identified through SEM-EDS analysis, is presented in Table 3. Generally, according to Table 3, the amount of nitrogen in the fiber surface area decreases as the concentration of the processing chemical increases. The results here are similar to the elemental analysis results (Table 2).

### 3.2. Epoxy Composites Characterization

**Figure 4:**
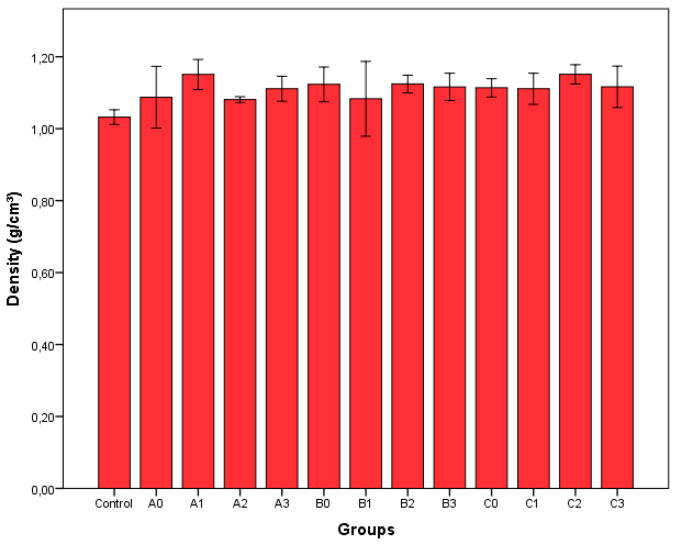
Densities of produced epoxy composites (treatment times: A = 20 min, B = 40 min, C = 60 min; ZnCl_2_ concentrations: 0 = 0%, 1 = 10%, 2 = 20%, 3 = 30%).

The density values of the produced epoxy composites filled with 1% recycled fibers groups are shown in Figure 4. Multivariate analysis of variance was performed to examine the effects of treatment time and ZnCl_2_ concentration on the values obtained. According to the results of the variance analysis, it was concluded that the changes in density were not significant at the 95% confidence interval (*p* = 0.05) according to treatment time (*p* = 0.739), ZnCl_2_ concentration (0.975), and the interaction of time and concentration (*p* = 0.445). According to Figure 4, it was determined that the densities varied between 1.03 g/cm^3^ and 1.15 g/cm^3^.

**Figure 5:**
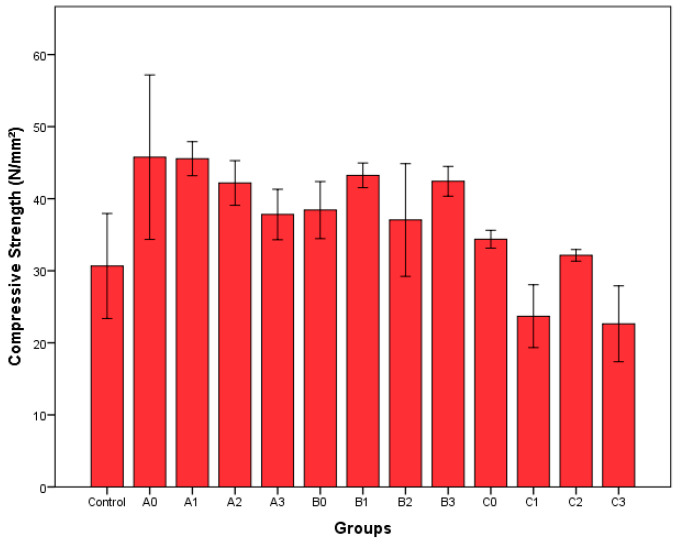
The average compressive strengths of produced epoxy composites (treatment times: A = 20 min, B = 40 min, C = 60 min; ZnCl_2_ concentrations: 0 = 0%, 1 = 10%, 2 = 20%, 3 = 30%).

The average compressive strengths of epoxy composites obtained by adding 1% recycled fibers as filler according to ASTM D 695-15 [33] are shown in Figure 5. Multivariate analysis of variance was performed to examine the effects of treatment time and ZnCl_2_ concentration on the values obtained. According to the results of the variance analysis, while the changes in compressive strength values were significant at the 95% confidence level for the treatment time (*p* = 0.00) and the interaction of treatment time and solution concentration (*p* = 0.49), it was found that the solution concentration alone did not affect the values (*p* = 0.218). According to Duncan’s analysis, the samples were divided into two groups according to the processing time (*p* ≤ 0.05). Group A consists of epoxy composites to which fibers were added 20 and 40 min after hydrolysis, while Group B consists of control and 60 min hydrolysis samples. In general, similar to our study, lignocellulosic fibers are known to increase the mechanical properties of epoxy composites [45]. According to Figure 5, although the values for the 60 min samples were lower than in the control group, it was found that these lows were not statistically significant. The decrease in the compression strength after 60 min is probably due to the change in curing behavior. This is because changes in particle size distribution affect properties of epoxy composites [23,36,37,46]. Studies in the literature, such as Potadar and Kadam (2018), have shown that tensile strength decreases as particle size increases [36], while Chauhan et al. (2021) reported in their study that tensile strength decreases especially as particle size decreases from 600 microns to 300 microns [37]. It is also known that chemical treatment of lignocellulosic materials improves the properties of epoxy composites [43]. Kacem et al. (2025) observed a decrease in mechanical properties with increasing acid concentration in the chemical treatment of yucca fibers [43]. Similarly, the decrease in compressive strength with increasing concentration and duration during the treatment can be said to be due to deterioration in the fibers caused by the acid effect.

**Figure 6:**
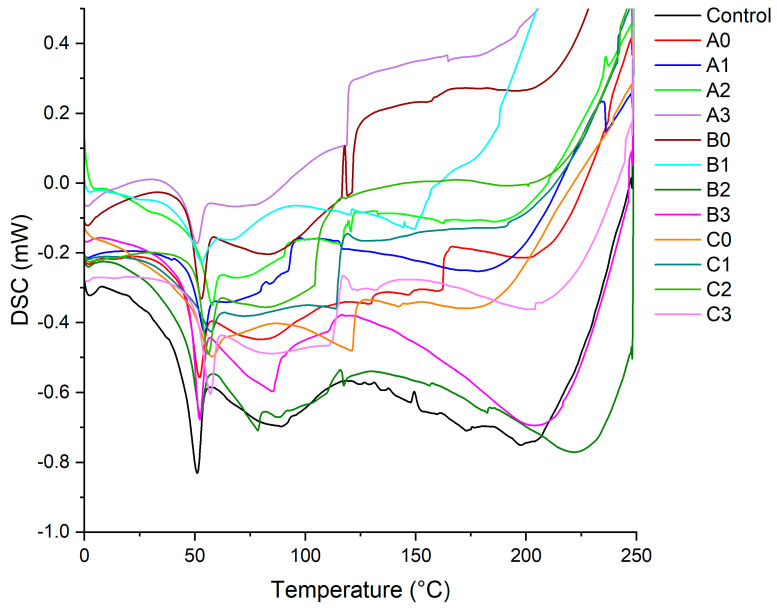
DSC analysis of epoxy composites (treatment times: A = 20 min, B = 40 min, C = 60 min; ZnCl_2_ concentrations: 0 = 0%, 1 = 10%, 2 = 20%, 3 = 30%).

The DSC analysis graph of epoxy composites containing 1% recycled fibers is shown in Figure 6. The graph shows that the Tg value of the composites with added recycled fibers increased and the ΔH (enthalpy) decreased. At this point, the change in both values indicates that the 1% recycled fibers increased the heat resistance of the epoxy composites. In general, the Tg values of the samples tended to increase with increasing hydrothermal recycling treatment time. This is thought to be due to the increase in the amount of fine fibers in the dimensional distribution shown in Figure 1. The increase in the amount of fine fibers likely affects the mobility of the chain segment of the macromolecules, resulting in a higher Tg value [47,48].

## 4. Conclusions

Based on the data acquired from the study, the following results can be concluded:

- ZnCl_2_ concentrations of 20% and 30% were effective in removing UF resin and resulted in a significant decrease in the nitrogen content of the recovered fibers. This situation was supported by FT-IR and SEM-EDS analyses.
- Time is more important than solution concentration in terms of dimensional changes. Long processing times (60 min) resulted in deterioration in fiber morphology and excessive particle size refinement, which negatively affected the compressive strength of epoxy composites.
- Composites produced with fibers added to epoxy at a 1% rate showed increased compressive strength. Fibers obtained by 20 min and 40 min hydrothermal hydrolysis can be used to improve the compressive strength of epoxy composites.
- When all measured values were evaluated together, it was determined that the optimal application parameters was 20 min of treatment at 30% ZnCl_2_ concentration.

ZnCl_2_ can have a potential use as a catalyst in the recycling of MDFs by hydrothermal hydrolysis. However, further studies are needed, such as the on properties of the obtained fibers and different optimization studies depending on where the fibers will be used.

## Figures and Tables

**Table 1:** Design of hydrothermal hydrolysis experiments.

Groups	Time (min)	Concentration (% *w*/*w*)
A0	20	-
A1	20	10
A2	20	20
A3	20	30
B0	40	-
B1	40	10
B2	40	20
B3	40	30
C0	60	-
C1	60	10
C2	60	20
C3	60	30

**Table 2:** Elemental compositions of recycled fibers.

Groups	N (%)	C (%)	H (%)
A0	1.376	46.204	5.945
A1	0.639	45.843	5.761
A2	0.397	43.426	5.686
A3	0.309	42.740	5.587
B0	1.166	46.601	5.694
B1	0.710	44.747	5.616
B2	0.334	44.630	5.429
B3	0.292	43.839	5.411
C0	1.337	46.716	5.665
C1	0.646	45.972	5.646
C2	0.365	44.912	5.511
C3	0.310	40.115	5.497

**Table 3:** SEM-EDS results of recycled fibers. (treatment times: A = 20 min, B = 40 min, C = 60 min; ZnCl_2_ concentrations: 0 = 0%, 1 = 10%, 2 = 20%, 3 = 30%).

	N		C		O	
Groups	Weight%	Atomic%	Weight%	Atomic%	Weight%	Atomic%
A0	6.57	6.32	53.41	59.95	40.02	33.72
A1	3.64	3.49	55.37	62.03	40.99	34.47
A2	1.99	1.89	59.66	66.18	38.35	31.93
A3	1.59	1.48	67.68	73.47	30.73	25.05
B0	7.49	7.08	59.87	65.94	32.64	26.98
B1	4.89	4.65	58.27	64.66	36.83	30.68
B2	4.26	4.04	59.68	66.02	36.05	29.94
B3	4.20	3.99	58.48	64.90	37.33	31.10
C0	3.84	3.59	65.40	71.25	30.76	25.16
C1	3.50	3.28	64.35	70.34	32.15	26.38
C2	2.87	2.70	62.82	69.00	34.31	28.30
C3	2.63	2.52	56.05	62.75	41.32	34.73

## Data Availability

The original contributions presented in this study are included in the article. Further inquiries can be directed to the corresponding author.

## Abbreviations

The following abbreviations are used in this manuscript:

MDF	Middle Density Fiberboard
UF	Urea—Formaldehyde
ZnCl_2_	Zinc chloride
FT-IR	Fourier transform infrared spectroscopy
SEM	Scanning Electron Microscope
EDS	Energy dispersive spectrometry
DSC	Differential scanning calorimetry
C	Carbon
N	Nitrogen
H	Hydrogen
O	Oxygen
A0	Recycled fiber hydrothermally treated for 20 min with pure water
A1	Recycled fiber hydrothermally treated for 20 min with 10% ZnCl_2_
A2	Recycled fiber hydrothermally treated for 20 min with 20% ZnCl_2_
A3	Recycled fiber hydrothermally treated for 20 min with 30% ZnCl_2_
B0	Recycled fiber hydrothermally treated for 40 min with pure water
B1	Recycled fiber hydrothermally treated for 40 min with 10% ZnCl_2_
B2	Recycled fiber hydrothermally treated for 40 min with 20% ZnCl_2_
B3	Recycled fiber hydrothermally treated for 40 min with 30% ZnCl_2_
C0	Recycled fiber hydrothermally treated for 60 min with pure water
C1	Recycled fiber hydrothermally treated for 60 min with 10% ZnCl_2_
C2	Recycled fiber hydrothermally treated for 60 min with 20% ZnCl_2_
C3	Recycled fiber hydrothermally treated for 60 min with 30% ZnCl_2_
